# Ringworms and Eczema

**Published:** 1891-06

**Authors:** 


### RINGWORMS AND ECZEMA.

The best remedy for ringworm depends entirely on the depth to which it has penetrated. When the disease is quite superficial it is very easily cured by painting the affected parts with strong acetic acid or tincture of iodine. But when the head is the point of attack, and the parasite gets deep down into the follicles of the hair, the difficulty is to get at it without so ruinous destruction of scalp as would be worse than ringworm. Under these circumstances, perhaps the best treatment for inveterate ringworm is to cut the hair short, and deal with a small patch at a time by means of croton oil; but as undiluted croton oil is very strong, no unpracticed hand should venture on its application. Next to this comes, perhaps, three daily annointings with compound citrine ointment. It sometimes happens that eczema supervenes on ringworm, and thus masks the original disease. The eczema must be treated first, and the ringworm dealt with afterwards. The ringworm is not really cured, but only disguised by the supervening eczema. It happens, also, that ringworm is followed by that disorder now called eczema impetiginodes, which some consider to be a combination of eczema and impetigo. It has very thick crusts, which must be removed and kept removed. I know a case which lasted for years, and underwent innumerable treatments, and at length, whether through treatment or spontaneously no one was quite sure, it disappeared. The hair must be cut short, the head well oiled, and then poulticed till the softened crusts can be shaled off. Wash the head with strained gruel, and when all the crusts are removed, apply benzoated zinc ointment, or some of the remedies which have been most recommended. After washing with gruel, always dry the head; wear a flannel cap, and persevere in whatever plan of treatment is adopted. The process of cure is sometimes tedious, but is accelerated by a general soundness of health, to which tonics are in many cases conducive.

